# TGFBR3 inhibits progression of papillary thyroid cancer by inhibiting the PI3K/AKT pathway and EMT

**DOI:** 10.1530/EC-24-0270

**Published:** 2024-11-21

**Authors:** Hanrong Zhang, Junxin Chen, Xin Chen, Chuimian Zeng, Pengyuan Zhang, Jiewen Jin, Haipeng Xiao, Yanbing Li, Hongyu Guan, Hai Li

**Affiliations:** 1 Department of Endocrinology and Diabetes Center, The First Affiliated Hospital of Sun Yat-sen University, Guangzhou, China; 2 Department of Endocrinology, Guizhou Hospital of the First Affiliated Hospital of Sun Yat-sen University, Guizhou, China

**Keywords:** TGFBR3, invasion, papillary thyroid cancer, PI3K/AKT pathway, EMT

## Abstract

**Background:**

Transforming growth factor beta receptor III (TGFBR3) has been shown to play a tumor-suppressive role in a variety of cancers. However, its role in papillary thyroid cancer (PTC) remains unknown.

**Method:**

TGFBR3 expression levels in PTC were analyzed utilizing The Cancer Genome Atlas (TCGA) and Gene Expression Omnibus (GEO) databases. Edu, wound healing, and Transwell assays were used to evaluate cell proliferation, migration, and invasion. Transcriptome sequencing, quantitative real-time reverse transcription-polymerase chain reaction (qRT-PCR), and Western blotting were used to detect the underlying mechanism of TGFBR3 in PTC progression.

**Result:**

This study demonstrated that TGFBR3 expression was significantly down-regulated in PTC compared to normal thyroid tissues. Low expression of TGFBR3 was associated with poor prognosis of patients with PTC. Furthermore, TGFBR3 expression positively correlated with thyroid differentiation score. In investigating the biological impact of TGFBR3 overexpression in PTC cell lines, we found that the proliferation, migration, and invasion of PTC cells were significantly inhibited in response to TGFBR3 overexpression. Moreover, we also demonstrated that overexpression of TGFBR3 inhibited the PI3K/AKT pathway and epithelial-mesenchymal transformation processes. Lastly, TGFBR3 expression was found to be involved in tumor immune infiltration, highlighting its potential influence on immune dynamics within the tumor microenvironment in PTC.

**Conclusion:**

TGFBR3 plays a tumor-suppressive role in PTC progression by inhibiting the PI3K/AKT pathway and epithelial mesenchymal transformation.

## Introduction

Thyroid cancer is the most common endocrine-related malignancy, with the seventh highest incidence among all cancers worldwide, whose incidence has increased significantly worldwide in recent decades (1, 2). The pathologic types of thyroid cancer include papillary thyroid cancer (PTC), follicular thyroid cancer (FTC), medullary thyroid cancer (MTC), and anaplastic thyroid cancer (ATC). PTC is the most common type, accounting for about 85% of cases (3). Although most PTC patients have a relatively good prognosis with a 98.5% five-year survival rate (4), some cases have high rates of distant organ or lymph node metastasis and recurrence, which is associated with poor prognosis (5, 6). The tumorigenesis and development of thyroid cancer involve multiple genetic and epigenetic alterations, among which the activation of MAPK and PI3K-AKT signaling pathways play fundamental roles in the molecular pathogenesis of thyroid cancer (7, 8). Additionally, other signaling pathways, such as transforming growth factor β (TGF-β), nuclear factor kappa-B (NF-κB), and Wnt/β-catenin signaling, also significantly contribute to the development and progression of thyroid cancer (7, 9). Considering the complexity of these pathways and their interactions, it is important to study how they affect the progression of PTC and the regulatory mechanisms involved.

Accumulating evidence has demonstrated the impact of TGF-β pathway activation in PTC. For instance, Brace et al. investigated the expression of TGF-β in thyroid nodules, revealing that TGF-β1 is significantly elevated in PTC compared to benign nodules, while TGF-β2 levels remain unchanged (10). Garcia-Rendueles revealed that the oncoprotein p27 alters TGF-β’s effects in PTC, leading to low proliferation and increased metastasis (9). Moreover, poorly circumscribed PTC is significantly associated with nodal metastases, especially in cases exhibiting high levels of TGF-β expression (11). However, the expression patterns of the TGF-β signaling pathway in PTC, its clinical significance, and its roles in disease progression are still not well understood.

TGF-β receptor 3 (TGFBR3) cannot phosphorylate, yet, it exhibits a strong binding capacity for all three variants of TGF-β. Moreover, the extracellular section of TGFBR3 can undergo a process of cleavage, resulting in a soluble extracellular fragment (sTGFBR3) which acts as a counteractive agent to TGF-β, blocking its interaction with TGFBR2. The intracellular segment of TGFBR3 is capable of interacting with other proteins (12). TGFBR3 has been identified as a tumor suppressor in a variety of cancers (13, 14, 15). Loss of TGFBR3 has been reported to promote the proliferation and metastasis of renal clear cell carcinoma (16). Another study showed that TGFBR3 can inhibit the proliferation and migration of bladder cancer cells (17). Other studies have shown that TGFBR3 plays a tumor-suppressive role in cervical cancer (18), head and neck cancer (19), lung cancer (20), breast cancer (21), etc. Currently, only a few studies have focused on the role of TGFBR3 in thyroid cancer, which merely identified TGFBR3 as a diagnostic or prognostic biomarker (22, 23). Our study aims to investigate the biological function of TGFBR3 in PTC and the underlying mechanisms.

## Materials and methods

### Cell culture

The PTC cell lines BCPAP, KTC1, and TPC1 were purchased from the Cell Bank of Type Culture Collection of the Chinese Academy of Sciences. The above cell lines have been verified by short tandem repeat (STR) DNA profiling (24), which were mycoplasma-free. These cells were cultured in Dulbecco’s modified Eagle’s medium (DMEM, Gibco, USA) supplemented with 10% fetal bovine serum (FBS, Gibco), streptomycin (100 mg/mL) (Gibco), and penicillin sodium (100 U/mL) (Gibco) at 37°C in a humidified atmosphere with 5% CO_2_.

### Patients and tissue collection

We collected the cancer and adjacent tissues of ten patients diagnosed with PTC pathologically. The samples were placed in liquid nitrogen immediately after excision for further investigation. Ethical approval from the Institutional Research Ethics Committee of the First Affiliated Hospital of Sun Yat-sen University and informed consent from all patients were obtained.

### Vectors and retroviral transfection

TGFBR3 overexpressing plasmid CMV-TGFBR3-Flag-SV40-eGFP-IRES-puro and empty control plasmid CMV-Flag-SV40-eGFP-IRES-puro were purchased from GeneCopoeia. BCPAP and KTC1 cells were infected with a lentivirus mixture prepared in advance for 48 h. The stable cell lines were selected with puromycin for 2 weeks. Western blotting was used to detect the protein expression level of TGFBR3 in transfected cells and evaluate the transfection efficiency.

### Transient trasfection

The siRNAs for TGFBR3 (TGFBR3-si-1, 5′-GGAGATGCTTCCCTGTTCA-3′; MMP9-si-2, 5′-GGGCCATGATGCAGAATAA-3′) were designed and synthesized by RiboBio Co. (Guangzhou, China). EndoFectin Max transfection reagent (GeneCopoeia, China) was used for transient transfection.

### RNA sequencing

Transcriptome sequencing and bioinformatics analysis of the samples were conducted by Shanghai Liebing Information Technology Co., LTD. Transcriptome sequencing was conducted by a commercial service (Novelbio, China). Total RNA was extracted from the samples using TRIzol reagent (Invitrogen) separately. The cDNA libraries were constructed for each RNA sample using the TruSeq Stranded mRNA Library Prep Kit (Illumina, Inc.) according to the manufacturer’s instructions. Purified first-strand cDNA was enriched by PCR to create the cDNA libraries. The libraries were quality-controlled with Agilent 2200 and sequenced by NovaSeq 6000 on a 150 bp paired-end run.

### Accession number

RNA sequencing data described in this article have been uploaded to the Gene Expression Omnibus (GEO) database. It can be accessed through GEO accession number GSE265794.

### RNA extraction and quantitative real-time polymerase chain reaction

Total RNAs were extracted using TRIzol (Invitrogen). cDNA synthesis was performed with 1 μg of total RNA as the template using GoScript™ Reverse Transcription Mix, Oligo (dT) (Promega, USA). Quantitative real-time reverse transcription-polymerase chain reaction (qRT-PCR) assay was performed on a QuantStudio™ 5 system. Specific primers were designed using Primer5.0 software and BLAST tested. All reactions were repeated three times, with GAPDH as the internal control. The relative expression of the indicated genes was calculated using the 2^−ΔΔct^ method. Primer sequences are listed in Supplementary Table 1 (see section on supplementary materials given at the end of this article).

### Western blotting

The indicated cells were lysed in radioimmunoprecipitation assay buffer (RIPA, Beyotime, China) supplemented with protease and phosphatase inhibitor cocktail (Epizyme, China). Bicinchoninic acid assay (BCA, Biosharp, China) was used to detect protein concentration. The proteins were separated using 10% SDS-PAGE (Epizyme) and transferred to a PVDF membrane (Millipore, USA). These membranes were blocked with 5% buttermilk at room temperature for 1 h prior to incubation with primary antibody. The immunoblot signal was visualized using the enhanced chemiluminescence (ECL) detection kit (Millipore, USA). The primary antibodies used are listed in Supplementary Table 2.

### EdU assay

The EdU incorporation assay was performed according to the manufacturer’s instructions (KeyGen BioTECH, China). The nuclei were stained with Hoechst 33342. Cells were observed under a fluorescence microscope (Olympus, Tokyo, Japan). The EdU-positive cell rate was measured by ImageJ software (NIH, USA). Each experiment was repeated three times.

### Wound-healing assay

The indicated cells were placed in six-well plates. When 90–95% confluence was achieved, the cells were scraped with the tip of the micropipette to create the wounds. The scraped cells were washed with PBS and incubated in a serum-reducing medium for 24 h. The wounds were observed and images were taken under a microscope. The Image-Pro Plus software was used to determine the distance between the wound edges. Each experiment was repeated three times.

### Transwell assay

The Transwell invasion and migration experiments were conducted using Transwell chambers (8 μm; Corning, USA), pre-coated with or without Matrigel (BD, USA) in a 24-well plate. The indicated cells were suspended in a serum-free DMEM medium. A 200 μL of single-cell suspension was added to the upper chamber, and 650 μL of medium containing 10% FBS was added to the lower cavity. After incubation for 24 h, the upper chambers were removed, and the cells on the surface of the upper chamber were gently wiped with a cotton swab. Cells migrating or invading to the other side were fixed with 4% paraformaldehyde, stained with 0.1% crystal violet, and counted under a microscope. Each experiment was repeated three times.

### Bioinformatic analysis

RNA sequencing profiles, clinical information, and survival data of the TCGA-THCA dataset were downloaded from The Cancer Gene Atlas (TCGA) database (25). The expression profiles of GSE33630, GSE60542, GSE29265, and GSE3467 were downloaded from GEO. The R package ‘ggplot2’ was used to visualize differential expression levels of TGFBR3 in PTC and normal thyroid tissue. Kaplan–Meier survival analysis was performed using the R package ‘survival’ and ‘survminer’. Gene Set Enrichment Analysis (GSEA) was performed using the R software package ‘clusterProfiler’. Thyroid differentiation score (TDS) and BRAF-RAS score (calculated by the correlations with the signature were used to drive a continuous measure (−1 to 1) with BRAF^V600E^-like being negative or RAS-like positive) and Risk score (estimated based on the 2009 American Thyroid Association guidelines) for samples in the TCGA-THCA dataset were downloaded from the supplementary materials of Nishant’s article (25, 26). All analyses were performed using the R software (v.4.2.1).

### Correlation analysis of TGFBR3 expression and tumor microenvironment in PTC

The correlation between TGFBR3 and tumor-infiltrating immune cells was analyzed by the Tumor Immune Estimation Resource (TIMER). CIBERSORT (http://cibersort.stanford.edu/) was used to analyze the infiltration of 22 immune cells in PTC. TGFBR3 was divided into two groups with low expression and high expression according to the median expression. According to the LM22 gene set of immune cells, the CIBERSORT deconvolution algorithm was used to calculate the relative abundance of 22 lymphocyte subtypes. The Wilcoxon test was used to compare the differences in the 22 immune cells between the two groups.

### Statistical analysis

The data were presented as mean ± s.d. Before testing for differences in the analyses of the current study, the normal distribution of the continuous variables was assessed by the Shapiro–Wilk test, and Levene’s test was applied for the evaluation of the homogeneity of variances. The Mann–Whitney and Kruskal–Wallis tests were used for analysis when the data were not normally distributed and exhibited non-homogeneity. For normal data with equal variance, we used the Student’s *t*-test or the ANOVA. Survival analysis was performed using Kaplan–Meier analysis and the Cox proportional hazards regression model. Cox proportional hazards regression was used to evaluate the association between TGFBR3 expression and disease-free survival (DFI) or progression-free survival (PFI) of PTC patients. The relationship between TGFBR3 and the clinical features of PTC patients was analyzed using *χ* ^2^ tests. Correlation analyses were performed using Pearson correlation tests. If not specified above, *P* < 0.05 was considered statistically significant. All statistical analyses were conducted using SPSS23 and R software (v.4.2.1).

## Results

### The expression of TGFBR3 is down-regulated in PTC

To explore the expression of genes involved in the TGF-β signaling pathway and their relationship with clinical outcomes in PTC, we downloaded TGF-β pathway-related genes from the PubChem website (https://pubchem.ncbi.nlm.nih.gov/pathway/tgfbrpathway). We then analyzed the expression differences of these genes between PTC and normal thyroid tissues using the TCGA-THCA cohort. This analysis identified five differentially expressed genes (log|FC| > 1, FDR < 0.05), namely, TGFBR1, TGFB1, TGFBR3, PPP1R15A, and CN2 (Supplementary Figure 1A). Subsequently, we conducted univariate Cox regression analysis to explore the association between these genes and the prognosis of PTC patients. As shown in Supplementary Figure 1B, we found that high TGFBR3 expression was significantly associated with favorable outcomes.

**Figure 1:**
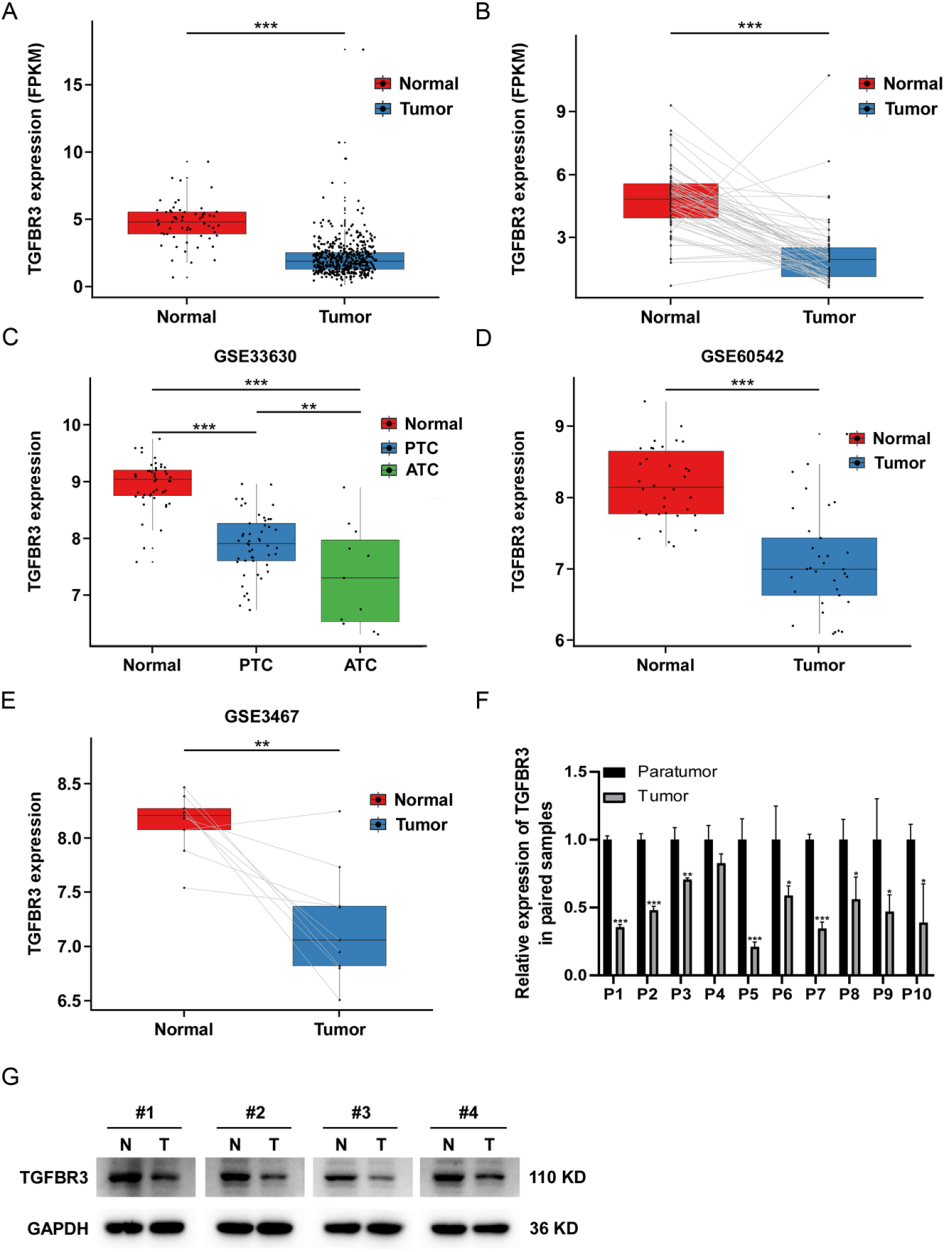
The expression of TGFBR3 is down-regulated in PTC. (A) The expression of TGFBR3 was lower in PTC tissues (*n* = 495) than in normal tissues (*n* = 59) in TCGA datasets. (B) The expression of TGFBR3 was lower in PTC tissues than in matched adjacent normal tissues in TCGA datasets (*n* = 59). (C) The expression of TGFBR3 in PTC tissues (*n* = 49), normal thyroid tissues (*n* = 45), and anaplastic thyroid cancer (*n* = 11) in GSE33630. (D) The expression of TGFBR3 in paired PTC (*n* = 33) and normal thyroid tissues (*n* = 24) in GSE60542. (E) The expression of TGFBR3 in paired PTC (*n* = 9) and matched adjacent normal tissues (*n* = 9) in GSE3467. (F) The mRNA expression of TGFBR3 in PTC tissues (*n* = 10) and paired adjacent normal tissues (*n* = 10) was detected by qRT-PCR. (G) The protein expression of TGFBR3 in PTC tissues (*n* = 4) and paired adjacent normal tissues (*n* = 4) was detected by Western blotting. Statistical analyses of Western blotting assays are shown in Supplementary Figure 2. Data are shown as mean ± s.d. *P < 0.05; **P < 0.01; ***P < 0.001. TGFBR3, transforming growth factor beta receptor III; PTC, papillary thyroid cancer.

To further investigate the expression of TGFBR3 in PTC, we analyzed the expression level of TGFBR3 in PTC using the TCGA database. Compared with normal thyroid tissue, TGFBR3 expression in PTC tissue was significantly down-regulated (Fig. 1A and B). To verify our finding, we analyzed the expression level of TGFBR3 using the GEO database. The results showed that the expression of TGFBR3 in PTC patients was also down-regulated compared with that in the normal control group (Fig. 1C, D, andE). We further collected ten pairs of PTC and its paired non-cancerous thyroid tissues and detected the expression of TGFBR3 by qRT-PCR. The mRNA expression level of TGFBR3 in PTC tissue samples was significantly lower than that in paired normal tissues (Fig. 1F). We performed WB analyses of TGFBR3 expression in four pairs of PTC and adjacent non-tumorous thyroid tissues. The results indicated that TGFBR3 protein levels in PTC tissues were significantly lower than those in matched normal tissues (Fig. 1G and Supplementary Figure 2). We further assessed the mRNA expression of TGFBR3 in 64 cases of PTC and analyzed its association with the clinical features of the patients. As shown in Supplementary Figure 3, there was a significant correlation between TGFBR3 expression and N classification and stage of PTC patients. Moreover, there was no significant correlation between TGFBR3 expression and gender or age of PTC patients (see Supplementary Figure 3).

### Low TGFBR3 expression in PTC patients is associated with poor prognosis and dedifferentiation

**Figure 2:**
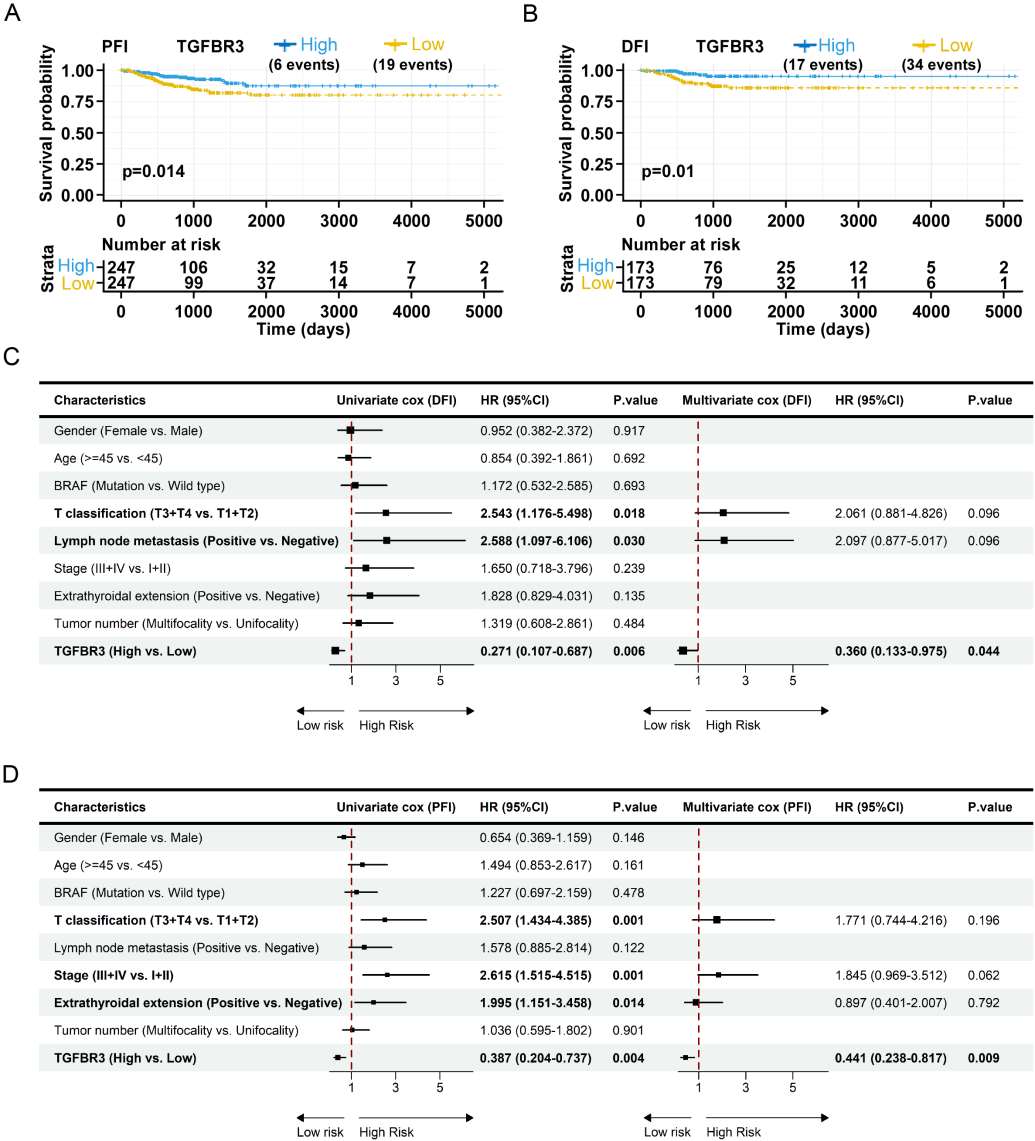
Low TGFBR3 expression is associated with clinical prognosis. PTC patients with high expression of TGFBR3 had a good prognosis in PFI (A) and DFI (B) revealed by Kaplan–Meier survival analysis. (C) Univariate and multivariate COX analysis of clinical features and DFI in PTC patients. (D) Univariate and multivariate COX analysis of clinical features and PFI in PTC patients. TGFBR3, transforming growth factor beta receptor III; PTC, papillary thyroid cancer; PFI, progression-free interval; DFI, disease-free interval.

To explore the relationship between TGFBR3 expression levels and prognosis of PTC patients, Kaplan–Meier survival analysis was conducted using the TCGA database. PTC patients were divided into high and low expression groups of TGFBR3 based on the median expression of TGFBR3. The results showed that downregulation of TGFBR3 was significantly associated with a shorter PFI and DFI (Fig. 2A and B). Patients with PTC have a favorable prognosis, and there are a limited number of deceased cases in the TCGA-THCA cohort, with only nine cases in the high TGFBR3 expression group and seven cases in the low TGFBR3 expression group for overall survival (OS) analyses. This is the same for disease-specific survival (DSS). Therefore, the statistical results may not be reliable due to the small sample size of deceased individuals in the cohort. Consequently, we did not analyze OS and DSS and focused only on DFI and PFI.

To further analyze the relationship between TGFBR3 expression levels and prognosis of PTC patients, we stratified PTC patients in the TCGA-THCA cohort at different TGFBR3 expression percentiles (25:75, 33:66, 50:50, 66:33, and 75:25) and performed analyses of DFI and PFI (Supplementary Table 3). Next, receiver operating characteristic (ROC) curves were drawn to determine the optimal cutoffs. Based on the achieved optimal cutoff value, PTC patients were divided into high and low expression groups of TGFBR3. The survival curves were plotted. In line with the results obtained using the median as a threshold, patients with high TGFBR3 expression demonstrated a significantly better prognosis compared to those with low expression (Supplementary Figures 4A and B).

Further analysis of the relationship between TGFBR3 expression and clinical characteristics in the TCGA-THCA cohort showed that there was a certain correlation between TGFBR3 expression and T classification, N classification, and TNM stage, but it was not statistically significant (Supplementary Table 4). We further used the TCGA-THCA dataset to conduct univariate analysis for DFI and PFI. The results showed that T classification and lymph node metastasis were risk factors for DFI, while high expression of TGFBR3 was a protective factor. Similarly, T classification, extrathyroidal invasion, and TNM stage were risk factors for PFI, while high expression of TGFBR3 was a protective factor. We also conducted multivariate Cox regression analysis, and the result indicated that low TGFBR3 expression was an independent prognostic predictor for both DFI and PFI for PTC patients (Fig. 2C and D).

**Figure 3:**
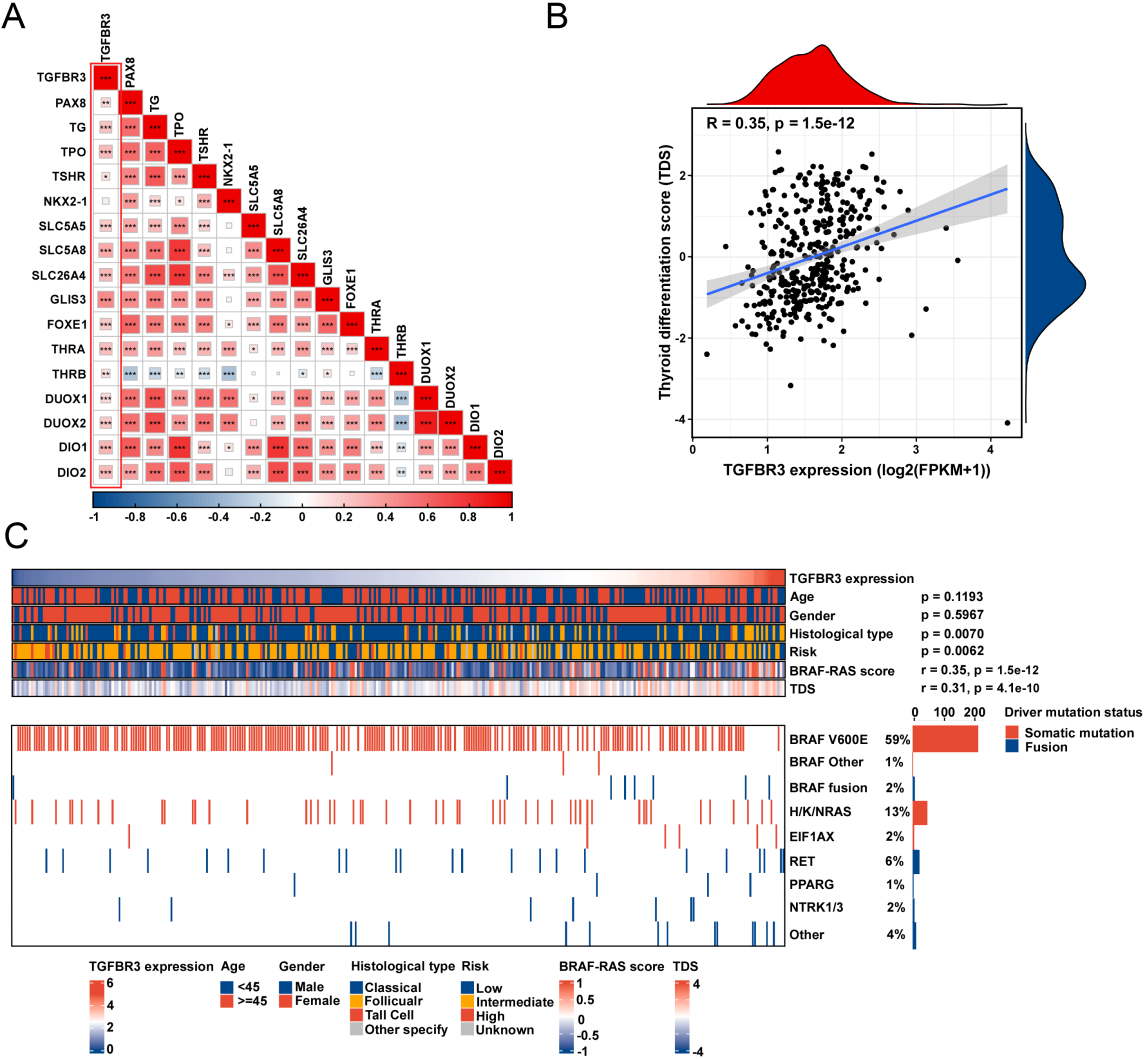
Relationship between TGFBR3 expression and thyroid differentiation and driver mutation events. (A) The correlation between TGFBR3 expression and the expression of thyroid differentiation-associated genes. (B) The relationship between TDS and TGFBR3 expression across samples from TCGA. (C) Heatmap of TGFBR3 expression and BRAF/RAS mutation status in TCGA PTC patients. Data are shown as mean ± s.d. *P < 0.05; **P < 0.01; ***P < 0.001; TGFBR3, transforming growth factor beta receptor III; PTC, papillary thyroid cancer; TDS, thyroid differentiation score.

We further investigated the correlation between TGFBR3 expression and PTC differentiation. We used the TCGA database to evaluate the correlation between TGFBR3 expression and the expression of thyroid differentiation-related genes (PAX8, TG, TPO, THSR, SLC5A5, SLC5A8, SLC26A4, GLIS3, FOXE1, THRA, THRB, DU). The results indicated that TGFBR3 levels were significantly positively correlated with the expression of these genes (Fig. 3A). Consistently, TGFBR3 expression positively correlated with thyroid differentiation score (TDS) (Fig. 3B).

To further investigate the driver mutation events related to TGFBR3, we analyzed the relationship between the main driver mutation events of PTC and TGFBR3 expression. As shown in Fig. 3C, TGFBR3 expression levels are negatively associated with recurrence risk, while positively correlated with BRAF-RAS score and TDS. Subsequently, we divided PTC patients into BRAF-like and RAS-like groups and analyzed the DFI and PFI of patients in both groups. No significant differences in DFI and PFI between PTC patients with high and low TGFBR3 expression were observed in both the BRAF-like and the RAS-like groups (Supplementary Figure 4C).

### TGFBR3 inhibits the proliferation, migration, and invasion of PTC cells

**Figure 4:**
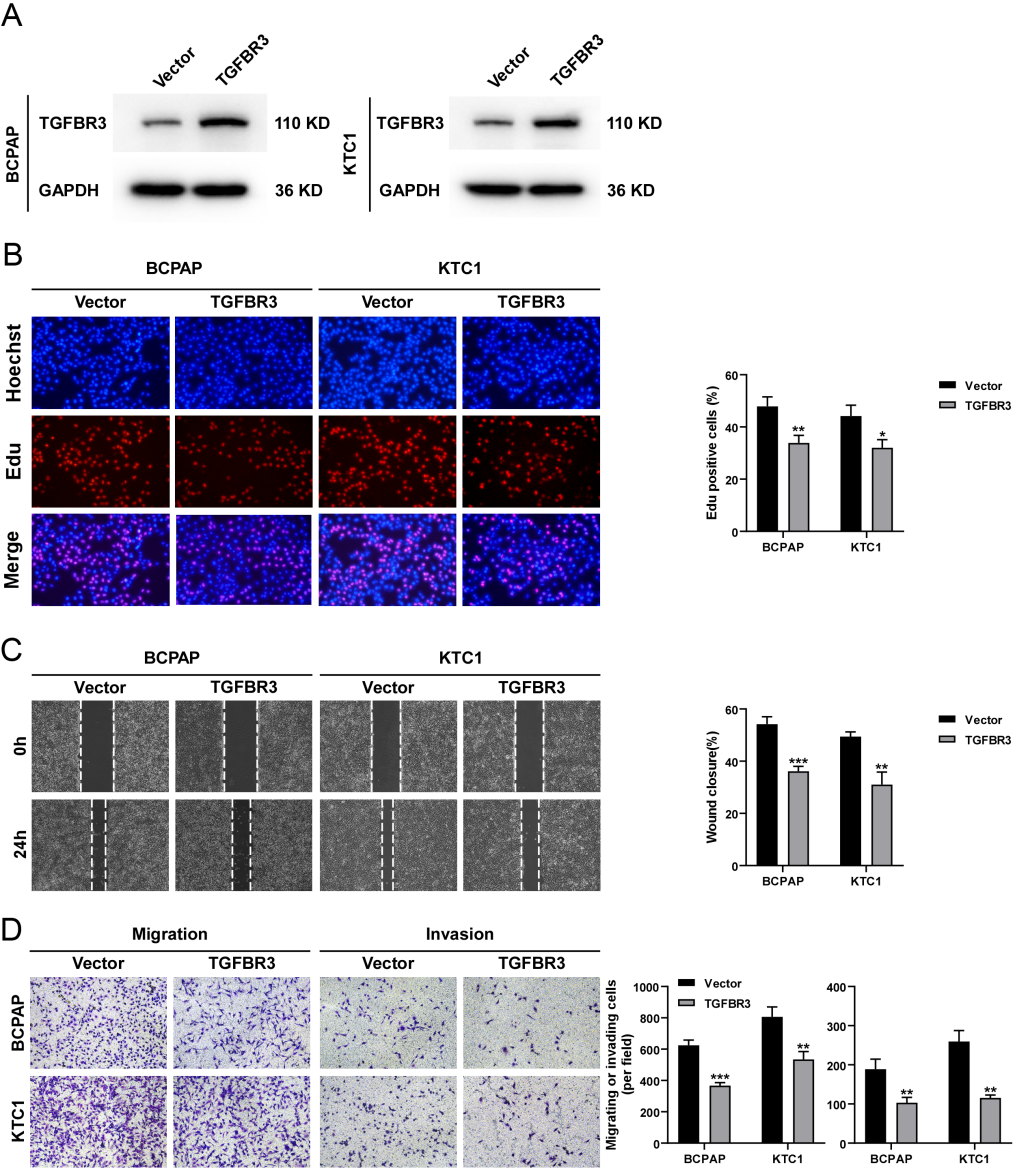
Effect of TGFBR3 overexpression on the biological function of PTC cells. (A) Overexpression of TGFBR3 in KTC1 and BCPAP cells was evaluated by Western blotting. (B) EdU assay showed that overexpression of TGFBR3 suppressed the proliferation of PTC cells. (C) Wound healing assay indicated that overexpression of TGFBR3 inhibited the migration of PTC cells. (D) Transwell assay indicated that overexpression of TGFBR3 suppressed the migration and invasion of PTC cells. Data are shown as mean ± s.d. Statistical analyses of Western blotting assays are shown in Supplementary Figure 6. *P < 0.05; **P < 0.01; ***P < 0.001; TGFBR3, transforming growth factor beta receptor III; PTC, papillary thyroid cancer.

We further investigated the effect of TGFBR3 overexpression on the biological function of PTC cell lines. We first used qPCR to measure the mRNA expression of TGFBR3 in the human thyroid epithelial cell line Nthy-ori3-1 and PTC cell lines including BCPAP, TPC1, KTC1, and IHH4. As shown in Supplementary Figure 5A, the results indicated that TGFBR3 was downregulated in PTC cell lines. Among the four PTC cell lines, TGFBR3 expression was lowest in BCPAP and KTC1 and highest in TPC1. We constructed BCPAP and KTC1 cell lines with stable overexpression of TGFBR3 (Fig. 4A and Supplementary Figure 6). We conducted EdU assays to evaluate the effect of TGFBR3 overexpression on the proliferation of PTC cells. The results showed that the percentage of EdU-stained cells in TGFBR3-overexpressing PTC cells was significantly reduced, indicating that TGFBR3 overexpression could inhibit the proliferation of PTC cells (Fig. 4B). Then we evaluated the effect of TGFBR3 overexpression on the migration ability of PTC cells through wound healing experiment. The results showed that overexpression of TGFBR3 significantly inhibited the migration ability of BCPAP and KTC1 (Fig. 4C). We further evaluated the effect of TGFBR3 overexpression on the migration and invasion ability of PTC cells by Transwell migration and invasion assays. The results showed that both BCPAP and KTC1 cells overexpressing TGFBR3 had significantly lower migration and invasion abilities (Fig. 4D). We further used siRNAs to knock down the expression of TGFBR3 in TPC1 (Supplementary Figure 7A) and performed EdU assays as well as Transwell assays. The results showed that the knockdown of TGFBR3 promoted the proliferative, migratory, and invasive abilities of TPC1 cells (Supplementary Figure 7B, C, and D).

### TGFBR3 inhibits PI3K/AKT pathway and epithelial-mesenchymal transformation in PTC cells

**Figure 5:**
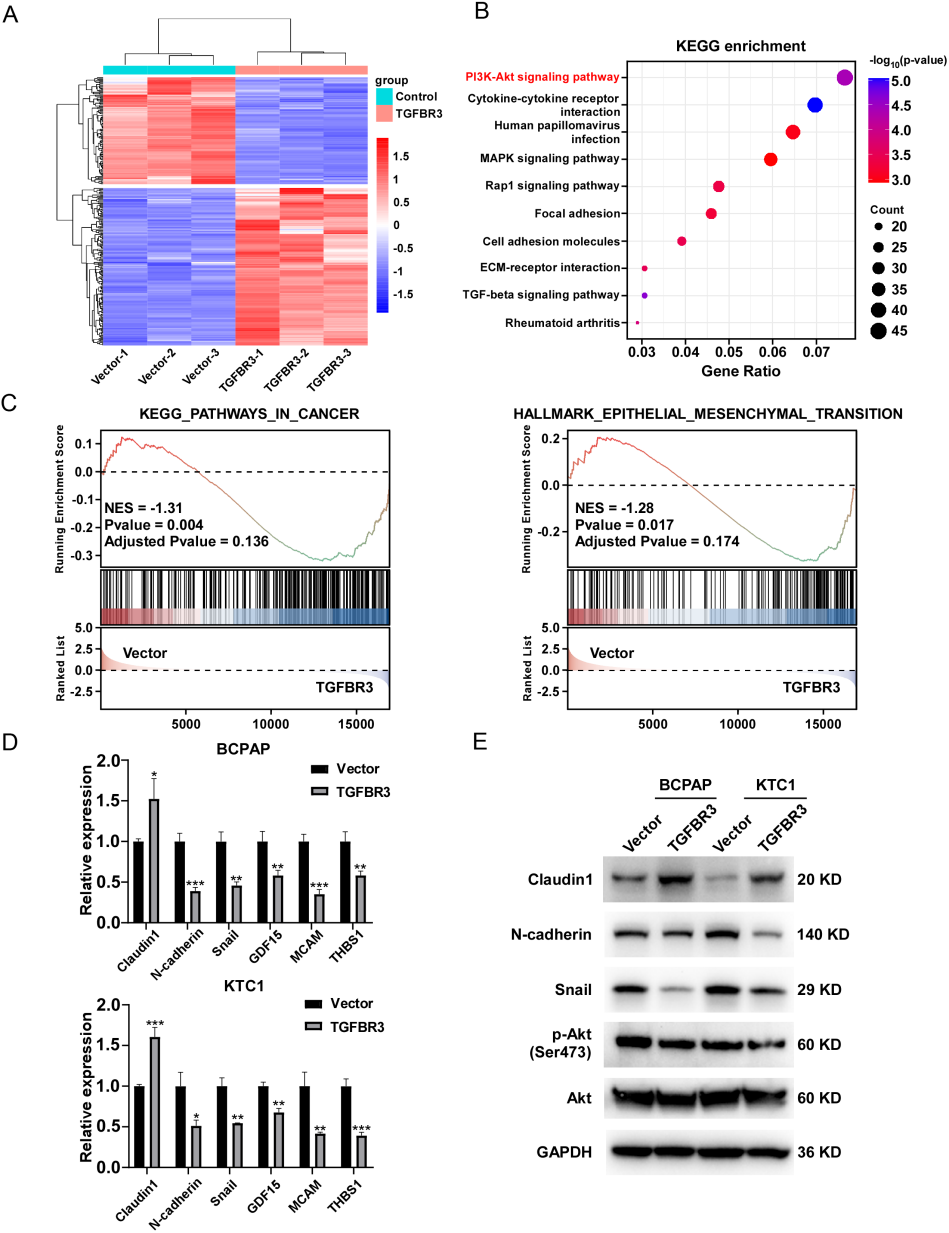
TGFBR3 is involved in regulating the PI3K/AKT pathway and EMT process in PTC. (A) Heatmap of differentially expressed genes between KTC1 cells overexpressing TGFBR3 and control vector based on RNA sequencing data. (B) Enrichment plot of the KEGG pathway analysis. (C) GSEA analysis of cancer and EMT pathways. (D) The mRNA expression levels of Snail, GDF15, THBS1, MCAM, N-cadherin, and Claudin1 in TGFBR3-overexpression KTC1 and BACAP cells and vector cells were detected by qRT-PCR. (E) The protein expression levels of Claudin1, N-cadherin, Snail, p-Akt, and Akt in TGFBR3-overexpression KTC1 and BACAP cells and vector cells were evaluated by WB. Data are shown as mean ± SD. Statistical analyses of Western blotting assays are shown in Supplementary Figures 10A and B. *P < 0.05; **P < 0.01; ***P < 0.001; KEGG, Kyoto Encyclopedia of Genes and Genomes; GSEA, gene set enrichment analysis; EMT, epithelial-mesenchymal transformation; TGFBR3, transforming growth factor beta receptor III.

To gain insight into how TGFBR3 regulates the biological function of TGFBR3, we further performed transcriptome sequencing on TGFBR3-overexpressing KTC1 cells and vector control cells. The expression of different genes in the two groups was shown as a heatmap. A total of 528 genes showed significant changes in their expression levels, among which 189 genes were down-regulated and 339 genes were up-regulated (|log2FC| ≥ 1, *P* < 0.05). The results showed that the expression of migration and invasion-related genes was downregulated in TGFBR3-overexpressing cells (FAT3, RUNX1T1, MMP19, LOXL4, SNAI1, SEMA5A, TGFB2, WNT2B, BMP4, CDH2, MCAM, GDF15, GFRA1, and THBS1, etc.) and up-regulated expression of the epithelial-related genes (CLDN1 and TG, etc.) (Supplementary Figure 8). The results of the Kyoto Encyclopedia of Genes and Genomes (KEGG) enrichment analysis showed that the expression of the PI3K/AKT pathway was inhibited after overexpression of TGFBR3 (Fig. 5A). We further performed GSEA analysis using the transcriptome sequencing data. The results showed that low expression of TGFBR3 was associated with cancer-related pathways and epithelial-mesenchymal transformation (EMT) in PTC cells (Fig. 5B).

We further verified the expression levels of EMT-related genes in TGFBR3-overexpressing and si-TGFBR3 PTC cells. qRT-PCR results showed that overexpression of TGFBR3 inhibited the expression of mesenchyme-related genes in BCPAP and KTC1 cells (Snail, GDF15, THBS1, MCAM, and N-cadherin), while it upregulated the expression of the epithelial-related gene Claudin1 (Fig. 5C). The opposite change was observed in si-TGFBR3 TPC1 cells (Supplementary Figure 9A). In order to further explore whether TGFBR3 inhibits the expression of the PI3K/AKT pathway and EMT-related proteins in PTC cells, we detected the expression of EMT and PI3K/AKT pathway-related proteins in BCPAP and KTC1 overexpressing TGFBR3 by Western blotting. The results showed that overexpression of TGFBR3 significantly decreased the phosphorylation level of AKT and the expression levels of N-cadherin and Snail, while increasing the expression levels of epithelial Claudin1 (Fig. 5D and Supplementary Figures 10A and B). The opposite trend was also observed in si-TGFBR3 TPC1 cells (Supplementary Figure 9B).

### Correlation between TGFBR3 expression and TME in PTC

**Figure 6:**
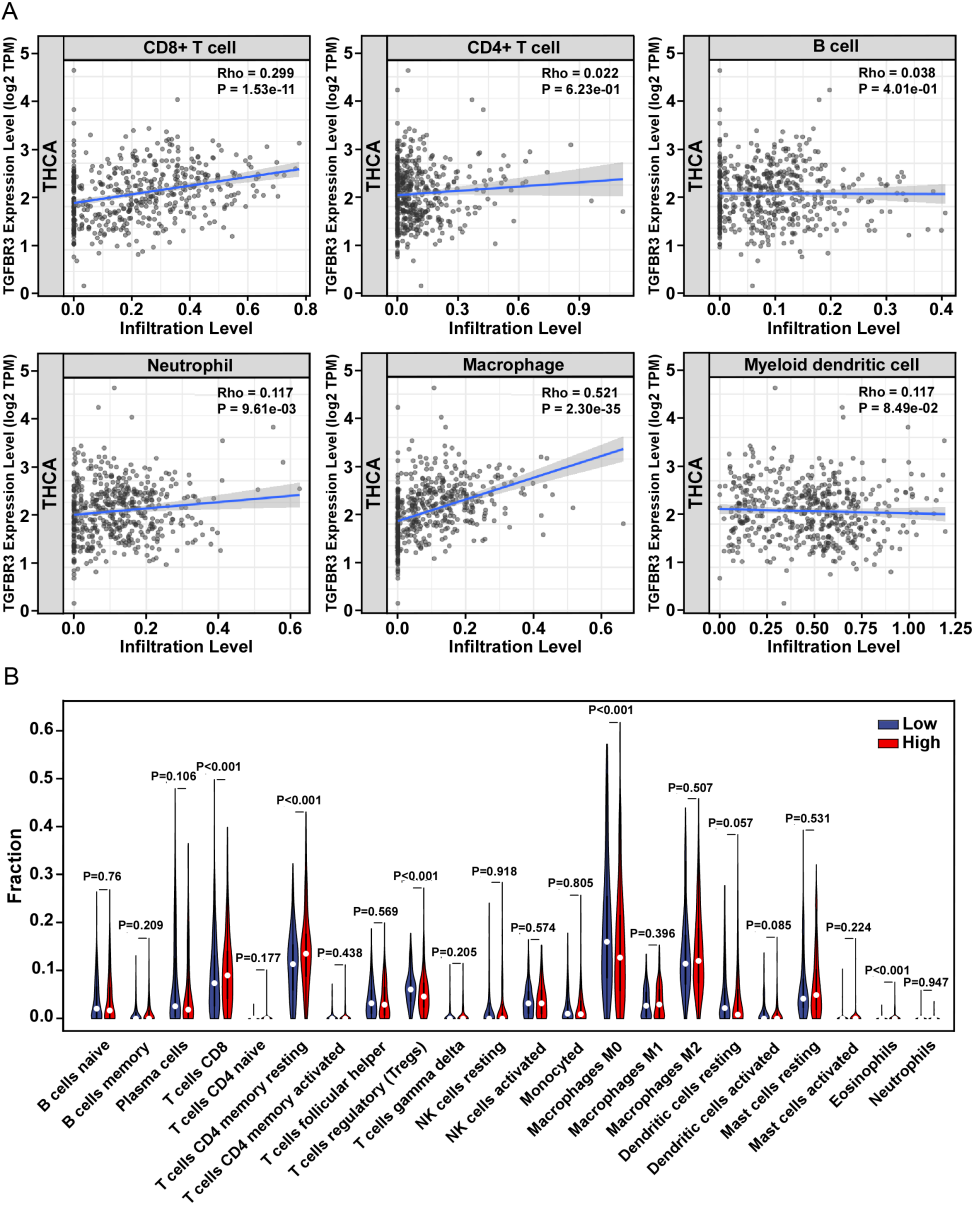
Association between TGFBR3 expression and TME. (A) The relationship between TGFBR3 expression and six types of immune infiltrates (B cells, CD8+ T cells, CD4+ T cells, dendritic cells, macrophages, and neutrophils) was analyzed by TIMER in PTC. (B) The correlation of TGFBR3 expression with immune cells infiltration were assessed by the data from CIBERSORT in PTC. TME, tumor immune microenvironment; TGFBR3, transforming growth factor beta receptor III; TIMER, Tumor Immune Estimation Resource; PTC, papillary thyroid cancer.

Growing evidence indicates that the immune microenvironment may have a crucial impact on PTC invasion (27). Therefore, we tried to find whether TGFBR3 expression was associated with immune infiltration in PTC. We first evaluated the correlation between TGFBR3 expression levels and tumor immunoinfiltration levels by using the tumor immune estimation resource (TIMER). The results showed that the expression level of TGFBR3 was positively correlated with the infiltration levels of B cells, CD4+ T cells, CD8+T cells, macrophages, and neutrophils in PTC, and negatively correlated with the infiltration levels of myeloid dendritic cells (Fig. 6A). We then used an established computational resource (CIBERSORT) to explore gene expression profiles of downloaded samples to infer the density of 22 types of immune cells. The results showed that the infiltration levels of M0 macrophages and regulatory T cells (Tregs) were significantly decreased in the high TGFBR3 expression group, while the infiltration levels of CD8+ T cells and CD4+ memory resting T cells were significantly increased (Fig. 6B).

## Discussion

In this study, we found that the expression of TGFBR3 is down-regulated in PTC patients, and low TGFBR3 expression is associated with poor prognosis. Cell experiments showed that TGFBR3 overexpression inhibited the proliferation, migration, and invasion of PTC cells by inhibiting EMT and the PI3K/AKT pathway. We also discovered that TGFBR3 is associated with tumor immune infiltration of PTC.

TGFBR3, as a co-receptor of the TGF-β family, inhibits the TGF-β pathway by binding to TGFβ1-3. In addition, TGFBR3 can also bind BMPs, GDF5, statin A, and statin B to increase or decrease the signal transduction of TGF-β family members (28, 29). The effect of TGFBR3 on various tumors and its influence on tumor progression is multifaceted. A study has shown that TGFBR3, regulated by miR-323b-3p, plays a role in tumor inhibition in the occurrence and development of osteosarcoma. Interestingly, their study also showed that TGFBR3 can activate EMT as a promoter of lung metastasis (30). Similarly, Liu *et al.* found that TGFBR3 plays a dual role in bladder cancer (31). Another study showed that the hsa_circ_0070100-hsa-miR-27a-3p/hsa-miR-27b-3p-TGFBR3 axis may play a regulatory role in the occurrence, development, migration, and survival of PTC (23). Down-regulation of TGFBR3 enhanced the malignant phenotype of esophageal squamous cell carcinoma (32). Our study found that TGFBR3 expression was down-regulated in PTC.

BRAF mutations, primarily occurring at codon V600, are the most common alterations in PTC and are found in approximately 60% of cases (25). The BRAFV600E mutation is linked to aggressive clinicopathologic features such as lymph node metastases, extrathyroidal extension, advanced stage, and unfavorable clinical outcomes (33, 34, 35). Our study found that the expression of TGFBR3 is positively correlated with BRAF-RAS score, while negatively associated with recurrence risk. The higher the degree of differentiation, the better the prognosis of thyroid cancer. Studies have shown that metastatic thyroid cancer with a high TDS score has a special response to radioactive iodine therapy (36). Consistent with our research, we showed that TGFBR3 expression level is positively correlated with TDS.

It has been reported in various types of cancer that EMT leads to increased tumor migration and aggressiveness. In the mechanism of tumorigenesis, the expression of the epithelial-related genes (such as E-cadherin and Claudins) is inhibited while the expression of mesenchymal-related genes (such as N-cadherin, Snail, ZEB, and Vimentin) is activated (37). The down-regulated expression of E-cadherin is associated with the migration and invasion of malignant tumors (38). Activation of N-cadherin can promote the metastasis of cervical cancer cells (39). The decreased expression of ZEB1 and N-cadherin inhibited the migration and invasion of BCPAP (40). HPIP knockout can inhibit the expression of N-cadherin in PTC and promote the expression of E-cadherin, thereby inhibiting EMT, proliferation, migration, and invasion of thyroid cancer *in vivo* and *in vitro* (41). Our study suggested that TGFBR3 could influence the EMT process in PTC progression. qRT-PCR showed that the expression of Snail, GDF15, THBS1, MCAM, and N-cadherin was down-regulated after overexpression of TGFBR3, and Claudin1 was up-regulated.

In addition, AKT also plays a crucial role in the regulation of EMT. It regulates EMT either through the non-classical Smad pathway of TGFβ or through the tyrosine kinase receptor or integrin-mediated AKT pathway. Downregulation of TGFBR3 expression can promote the EMT process of liver cancer by inducing activation of the SMAD2 and AKT pathways, thus promoting its progression (42). CircNDST1 can promote the interaction between CSNK2A1 and AKT, activate the PI3K/AKT pathway and EMT, and affect the migration and invasion ability of the KTC1 cell line (43). Our study showed that overexpression of TGFBR3 could down-regulate the protein expressions of N-cadherin and Snail1 during EMT, and suppress the expression levels of phosphorylated PI3K/AKT and Claudin1. But whether TGFBR3 regulates the EMT process through PI3K/AKT in PTC needs to be further explored.

In terms of tumor progression, the influence of the tumor microenvironment (TME) on PTC should not be underestimated. EMC remodeling, apoptosis, angiogenesis, and EMT are simultaneously involved in the occurrence and development of thyroid cancer in TME (44). Our sequencing results also showed that many differentially expressed genes were mainly related to tumor invasion and metastasis, apoptosis, and angiogenesis, and overexpression of TGFBR3 could inhibit the expression of the above-related genes, including FAT3, RUNX1T1, MMP19, LOXL4, SNAI1, SEMA5A, TGFB2, WNT2B, BMP4, CDH2, MCAM, GDF15, GFRA1, and THBS1, etc. Among them, GDF15 is significantly down-regulated in the TGFBR3 overexpression group, which is consistent with previous studies reporting that GDF15-induced STAT3 can promote thyroid cancer invasion (45). THBS1 expression has been shown to be associated with poor prognosis in medullary thyroid carcinoma (46). In our results, overexpression of TGFBR3 inhibits the expression of these genes.

In summary, we illustrated that TGFBR3 is downregulated, and low expression of TGFBR3 correlates with a worse prognosis in PTC. Overexpression of TGFBR3 inhibited the proliferation, migration, and invasion of PTC cells. TGFBR3 may inhibit the progression of PTC by inhibiting the EMT and PI3K/AKT pathways, which play a tumor suppressor role in PTC. Our data provide new insights into the pathogenesis of PTC and offer a potential target for the treatment of the disease.

## Supplementary Materials

Supplementary Material

## Declaration of interest

The authors declare that there is no conflict of interest that could be perceived as prejudicing the impartiality of the research reported.

## Funding

This research was supported by grants from the Guangzhou Science and Technology Projects (2023A04J2190).

## Ethical approval

The Ethics Committee approval was obtained from the Ethics Committee of the First Affiliated Hospital of Sun Yat-sen University.

## Data availability statement

The datasets analyzed for this study can be found in the TCGA (https://cancergenome.nih.gov/), GSE33630 (https://www.ncbi.nlm.nih.gov/geo/query/acc.cgi?acc=GSE33630), GSE60542 (https://www.ncbi.nlm.nih.gov/geo/query/acc.cgi?acc=GSE60542), GSE29265 (https://www.ncbi.nlm.nih.gov/geo/query/acc.cgi?acc=GSE29265) and GSE3467 (https://www.ncbi.nlm.nih.gov/geo/query/acc.cgi?acc=GSE3467).

## Author contribution statement

Hanrong Zhang; Junxin Chen: Performed the experiments; Analyzed and interpreted the data; Wrote the paper. Xin Chen; Chuimian Zeng; Pengyuan Zhang; Jiewen Jin: Performed the experiments; Wrote the paper. Haipeng Xiao; Yanbing Li: Conceived and designed the experiments. Hongyu Guan; Hai Li: Conceived and designed the experiments; Analyzed and interpreted the data; Contributed reagents, materials, analysis tools, or data; Wrote the paper.
