# Supplementary material for: TGFBR3 inhibits progression of papillary thyroid cancer by inhibiting the PI3K/AKT pathway and EMT
Source: Endocr Connect. 2024 Nov 21;13(12):e240270. doi: 10.1530/EC-24-0270 (PMC11623029; doi:10.1530/EC-24-0270)
Supplement: Supplementary Material [file supplementary_material.pdf]

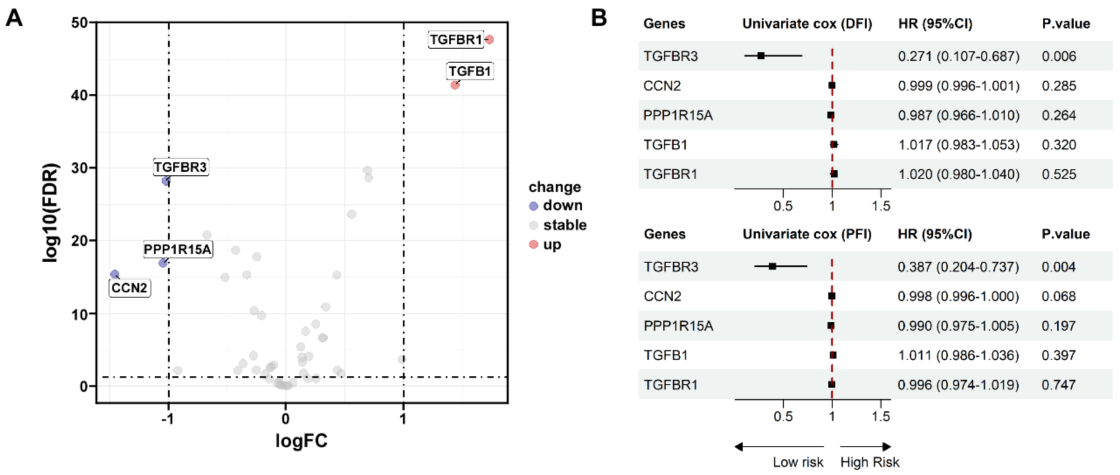

Supplementary Figure 1. (A) Heatmap showing the differential expression of TGF-β pathway-related genes between PTC and normal thyroid tissues using the TCGA-THCA cohort (log|FC| > 1, FDR < 0.05). (B) Univariate Cox regression analysis was used to explore the association between the differentially expressed and the prognosis of PTC patients. TGFBR3 was significantly associated with favorable outcomes. PTC, papillary thyroid cancer.

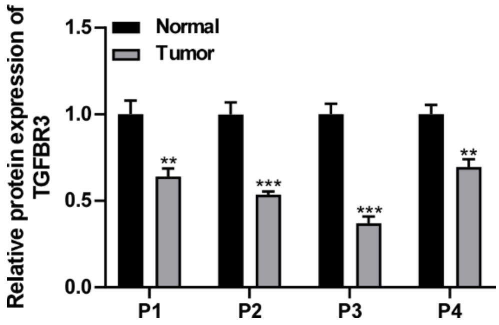

Supplementary Figure 2. Statistical analyses of Western blotting assays in Fig. 1G. *, P < 0.05; **P < 0.01; ***P < 0.001; #, p > 0.05.

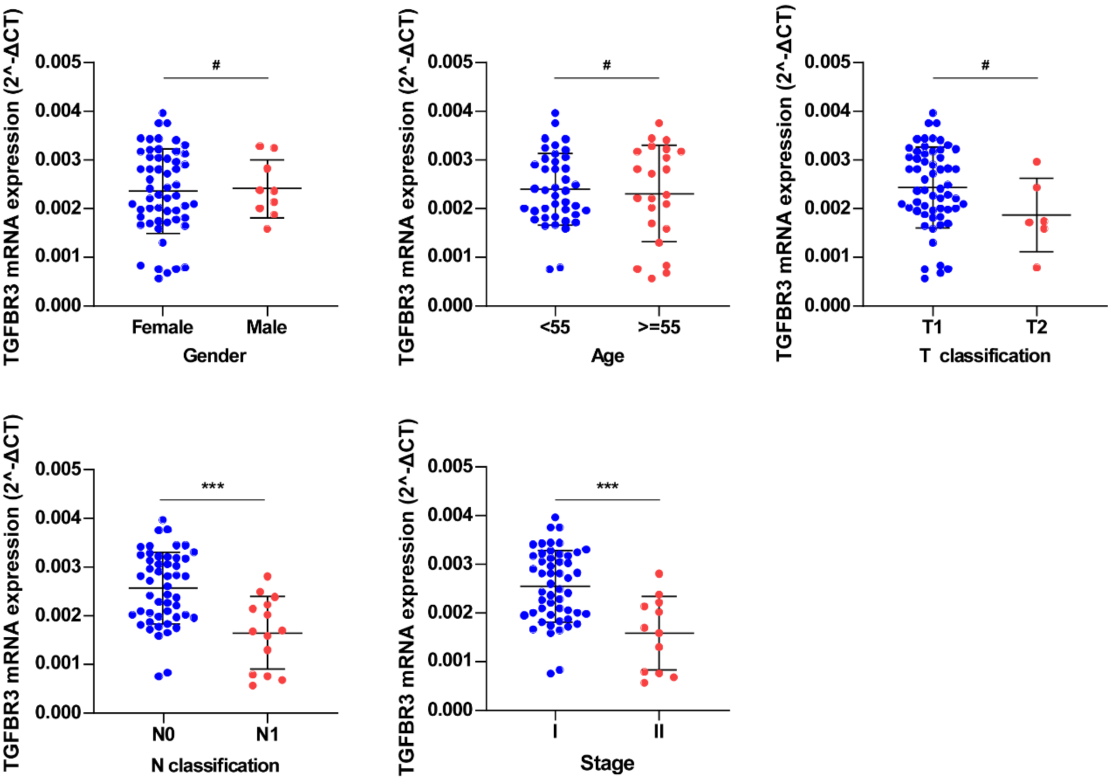

Supplementary Figure 3. Correlation between TGFBR3 mRNA expression and the clinical features of PTC patient. *, P < 0.05; **P < 0.01; ***P < 0.001; #, p > 0.05.

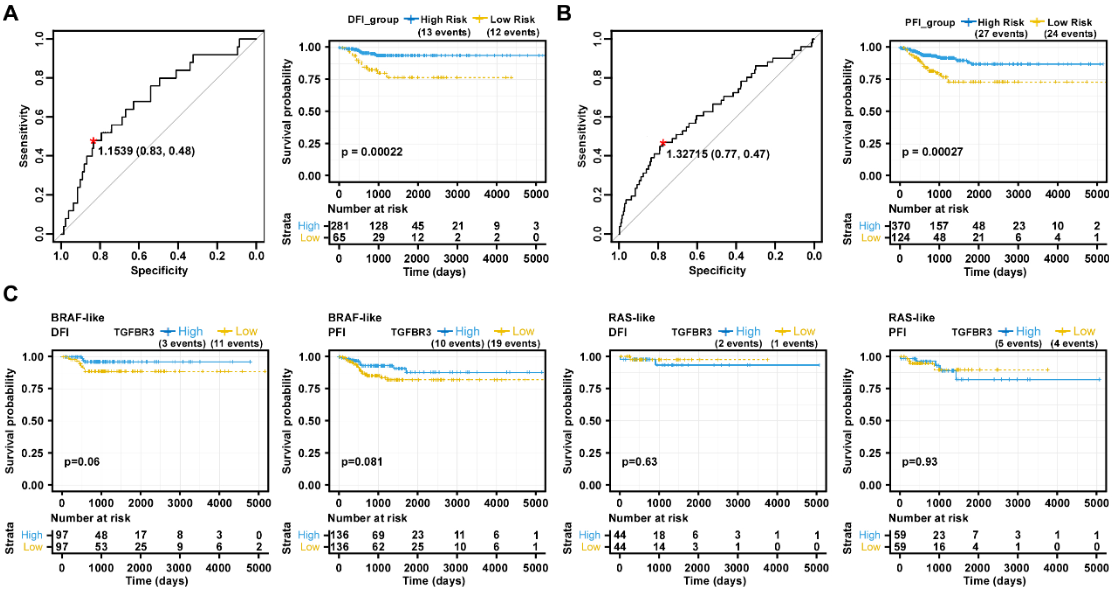

Supplementary Figure 4. (A, B) Receiver operating characteristic (ROC) curves were drawn to identify optimal cutoffs. Kaplan-Meier analyses of DFI and PFI of PTC patients were performed. PTC patients were divided into high and low expression groups of TGFBR3 based on the optimal cutoffs. (C) PTC patients were divided into BRAF-like and RAS-like groups. Kaplan-Meier

analyses of DFI and PFI in both groups were performed. DFI, disease-free interval; PFI, progression-free interval; PTC, papillary thyroid cancer.

A

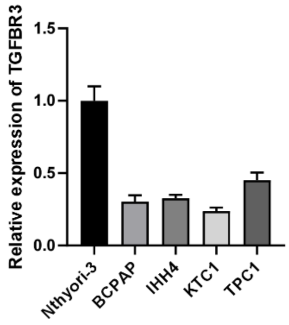

B

Cell line	Key oncogenic mutations
BCPAP*	BRAF p.V600E
IHH4	BRAF p.V600E
KTC1*	BRAF p.V600E
TPC1	RET/PTC1 fusion

Supplementary Figure 5. (A) TGFBR3 mRNA expression in thyroid epithelial cell line Nthyori-3 and thyroid cancer cell lines BCPAP, IHH4, KTC1 and TPC1 was detected via qRT-PCR. (B) Key oncogenic mutations of PTC cell lines. *, poorly differentiated papillary thyroid cancer.

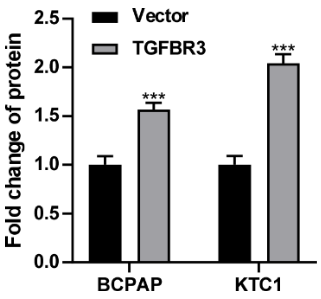

Supplementary Figure 6. Statistical analyses of Western blotting assays in Fig. 4A. *, P < 0.05; **P < 0.01; ***P < 0.001; #, p > 0.05.

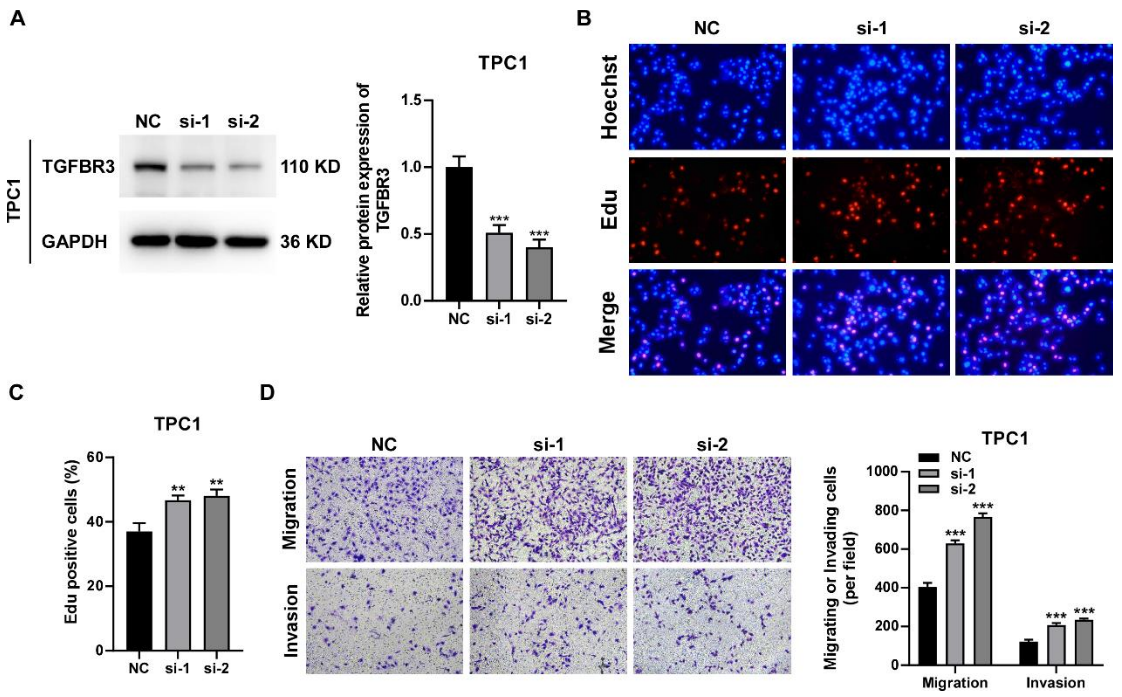

Supplementary Figure 7. Effect of knocking down TGFBR3 on the biological function of PTC cell line TPC1. (A) Knocking down of TGFBR3 in TPC1 was evaluated by Western Blotting. (B, C) EdU assay showed that knocking down of TGFBR3 promoted the proliferation of TPC1. (D) Transwell assay indicated that knocking down of TGFBR3 suppressed the migration and invasion of TPC1. *, P < 0.05; **P < 0.01; ***P < 0.001; #, p > 0.05.

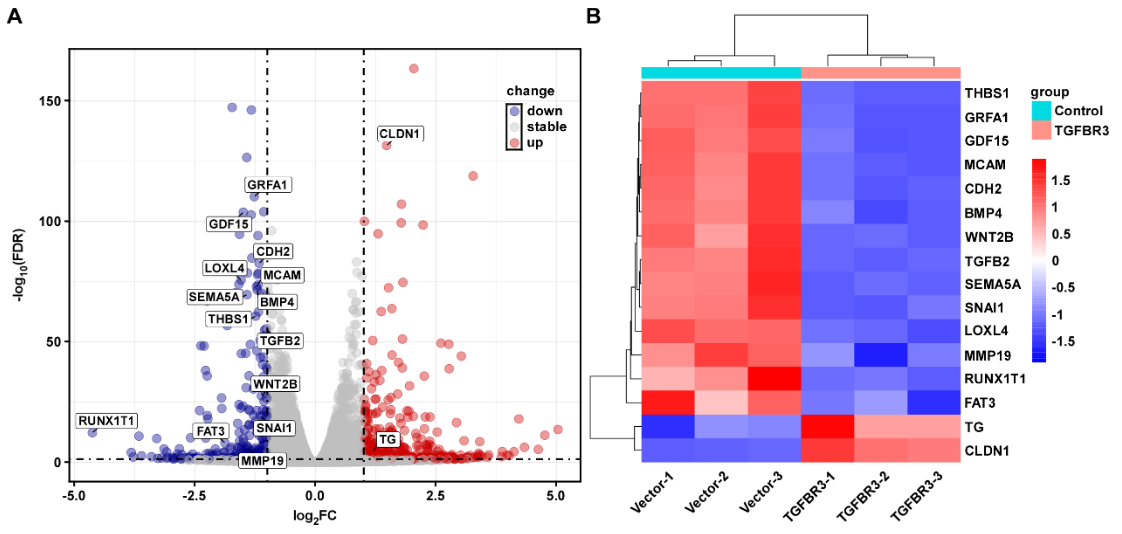

Supplementary Figure 8. Volcano map (A) and heatmap (B) indicating the down-regulated migration and invasion related genes in TGFBR3-overexpressing cells and up-regulated

epithelial-related genes based on the result of RNA sequencing.

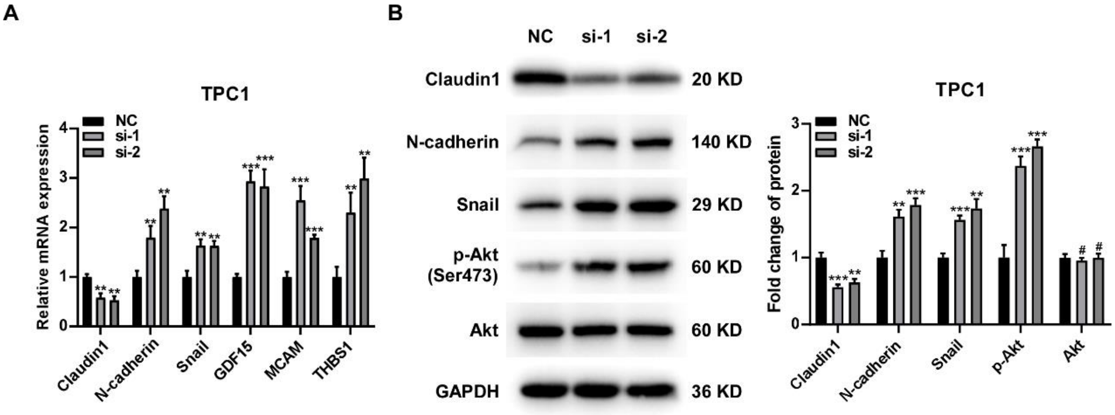

Supplementary Figure 9. Effect of knocking down TGFBR3 on PI3K/AKT pathway and EMT of PTC cell line TPC1. (A) The mRNA expression levels of Snail, GDF15, THBS1, MCAM, N-cadherin and Claudin1 in si-TGFBR3 TPC1 cells were detected by qRT-PCR. (B) The protein expression levels of Claudin1, N-cadherin, Snail, p-Akt and Akt in si-TGFBR3 TPC1 cells were evaluated by WB. *, P < 0.05; **P < 0.01; ***P < 0.001; #, p > 0.05.

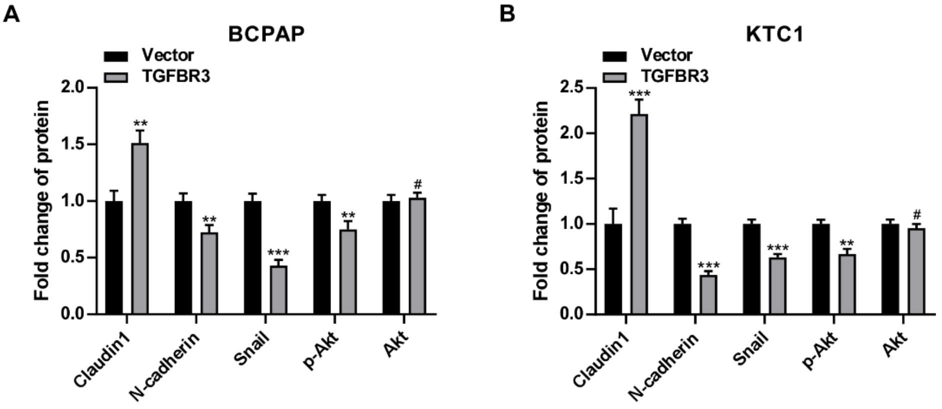

Supplementary Figure 10. Statistical analyses of Western blotting assays in Fig. 5D. *, P < 0.05; **P < 0.01; ***P < 0.001; #, p > 0.05.

Supplementary Table 1. Primers used in the experiment

Gene	Primer sequencing
GAPDH	Forward primer：GAAAGCCTGCCGGTGACTAA
	Reverse primer：GCCCAATACGACCAAATCAGAG
ACTB	Forward primer：ACAGAGCCTCGCCTTTGC
	Reverse primer：GATATCATCATCCATGGTGAGCTGG
TGFBR3	Forward primer：ACCTGAAATCGTGGTGTTT
	Reverse primer：AAGGTGATGTTTCCGTGGG
Snail1	Forward primer：GGCCTAGCGAGTGGTTCTTC
	Reverse primer：GCTGCTGGAAGGTAAACTCTGG
THBS1	Forward primer：CAGGAGCAACCTCTACTCCG
	Reverse primer：CAGCAGGGATCCTGTGTGT
GDF15	Forward primer：ATACTCACGCCAGAAGTGCG
	Reverse primer：TCTTGCAAGGCTGAGCTGAC
MCAM	Forward primer：AACAGCACCTCCACAGAGAG
	Reverse primer：TCTTACGAGACGGGGGTAGC
N-cadherin	Forward primer：GGCGTTATGTGTGTATCTTCACTG
	Reverse primer：GCAGTTGCTAAACTTCACTGAAAGG
Claudin1	Forward primer：CCAGTCAATGCCAGGTACGA
	Reverse primer：GCTGGAAGGTGCAGGTTTTG

Supplementary Table 2. The primary antibody used in the experiment

Antibody	Company (RRID, Size)	Working dilutions
Anti-TGFBR3	Cell Signaling (AB_10698740, 100 µl)	1:1000
Anti-p-AKT	Cell Signaling (AB_2315049, 100 µl)	1:2000
Anti-Akt	Cell Signaling (AB_915783, 100 µl)	1:1000
Anti-claudin1	Abcam ((AB_3082989, 40 µl)	1:2000
Anti-Snail	Proteintech (AB_2191756, 50 µl)	1:500
Anti-N-cadherin	Proteintech (AB_2881610, 50 µl)	1:5000
Anti-GAPDH	Proteintech (AB_2107436, 50 µl)	1:50000

Supplementary Table 3. Survival analysis of patients stratified at different TGFBR3 expression percentiles based on TCGA-THCA cohort

Survival	Stratification (%)	p value
DFI	25: 75	0.00348
	33: 66	0.02447
	50: 50	0.01041
	66: 33	0.06260
	75: 25	0.03907
PFI	25: 75	0.00027
	33: 66	0.00998
	50: 50	0.01439
	66: 33	0.02059
	75: 25	0.03660

DFI, disease-free interval; PFI, progression-free interval.

Supplementary Table 4. Correlation between TGFBR3 expression level and clinical features of PTC patients in TCGA

Clinicopathologic variables (n)	TGFBR3		p value
	Low	High	
Age， years			
<45 (223)	104	119	0.3341
≥45 (272)	143	129	
Gender			
Male (130)	59	71	0.3579
Female (365)	188	177	
TNM stage			
I and II (328)	153	175	0.0887
III and IV (165)	94	71	
Unknown (2)			
T classification			
T1 and T2 (307)	139	168	0.0870
T3 and T4 (186)	107	79	
Unknown (2)			
Lymph node metastasis			
No (226)	110	116	0.0921
Yes (219)	119	100	
Unknown (50)			
